# BCG Vaccination in HIV-Infected Children

**DOI:** 10.1155/2011/712736

**Published:** 2011-04-10

**Authors:** James J. C. Nuttall, Brian S. Eley

**Affiliations:** Paediatric Infectious Diseases Unit, Red Cross Children's Hospital and University of Cape Town, Rondebosch 7700, South Africa

## Abstract

Despite the use of Bacillus Calmette-Guérin (BCG) vaccination for many years, infants and young children exposed to adults with infectious forms of tuberculosis (TB) are at high risk of developing complicated TB disease. This risk is much higher among HIV-infected children, and data on BCG protective efficacy in HIV-infected children is lacking. Recent research on BCG safety in HIV-infected infants has resulted in policy shifts, but implementation is challenging. New approaches to preventing TB among infants and children, particularly HIV-infected infants, are needed. This paper briefly reviews BCG safety and efficacy considerations in HIV-infected infants and discusses other approaches to preventing TB, including new TB vaccines and vaccination strategies.

## 1. Introduction

It is estimated that worldwide over 100 million doses of BCG vaccine are administered per year [1]. However, the exact nature of protection against TB disease following BCG vaccination has not been fully elucidated. Vaccine efficacy and safety in the context of HIV infection is controversial and is a topic of considerable recent and ongoing research. Such research is highly relevant to global public health as TB endemic areas frequently overlap with areas of high HIV prevalence, and HIV/TB coinfection is common amongst both adults and children. Between 2004 and 2006, the incidence of TB in HIV-infected infants in Cape Town, South Africa was reported as 1596 per 100 000 compared to 66 per 100 000 in HIV-uninfected infants [2]. Infants and young children exposed to adults with TB are at high risk for developing severe and complicated forms of TB disease. Diagnostic difficulties and complexity related to the clinical management of coinfected infants and children contribute to the scale of the paediatric problem. There is an urgent need for safe and effective TB prevention strategies in infants and children, particularly those who are HIV-infected.

## 2. BCG Efficacy

The clinical efficacy of a vaccine is measured in terms of the percentage of reduction in disease among vaccinated individuals that is attributable to vaccination. Efficacy represents a composite outcome of three events: (1) the proportion of vaccine “take”, (2) the degree of vaccine protection, and (3) the duration of protective immunity [3]. 

Research on BCG efficacy has been hampered by the failure to identify a reliable clinical or immunological parameter that indicates protection against the development of TB disease following BCG vaccination. Neither the presence of a BCG scar nor tuberculin skin test reactivity correlates with protection against TB [4]. 

In an attempt to better understand BCG-induced immunity, investigators have described the nature of immune responses following BCG vaccination and latent TB infection. In many individuals, BCG vaccination is capable of inducing a polyfunctional T cell response dominated by cells capable of producing type 1 cytokines (including interferon- (IFN-) gamma, interleukin- (IL-) 2 and tumour necrosis factor (TNF)) [5]. 

There have also been attempts to delineate specific immune defects in individuals who are highly susceptible to developing TB disease or complications following vaccination with live, attenuated BCG, including HIV-infected individuals and infants with primary immunodeficiency disorders [3]. Among HIV-infected infants not receiving antiretroviral therapy, BCG has been shown to induce a much lower frequency and quality of specific CD4 T cells than in HIV-uninfected infants, and this persists throughout the first year of life [6]. Six-week-old HIV-exposed (born to HIV-infected mothers) but uninfected infants who had received BCG at birth had lower IFN-gamma responses than HIV-unexposed infants [7]. In children receiving antiretroviral therapy, antimycobacterial immune responses may be restored [8]. Individuals with genetic disorders of the IFN-gamma cytokine pathway are particularly predisposed to developing active TB disease [9]. 

Large randomized clinical trials have provided data on BCG efficacy. In the HIV-uninfected infants, administration of a single dose of Bacillus Calmette-Guérin (BCG) vaccine at or soon after birth has been shown to afford approximately 80% protection against disseminated forms of TB (miliary TB and TB meningitis) in infancy [10–12]. BCG is less effective and variable in preventing pulmonary TB in infants and adults with efficacies between 0% and 80% reported in clinical trials during the last century [12]. These findings suggest that BCG is unlikely to be effective in preventing infection with *M. tuberculosis* but may prevent progression of infection to disease, particularly disseminated forms of TB disease. Since disseminated disease is more common amongst young children and is associated with high morbidity and mortality, BCG vaccination of newborns may be cost-effective as a paediatric intervention but has little impact on transmission of TB amongst adults [3]. Factors believed to influence the variable protection include BCG strain variation, vaccine dose, patient age at vaccination, nutritional status, host genetic factors, environmental mycobacterial and helminthic infections, and geographic location [13–15]. 

Data on the protective efficacy of BCG amongst HIV-infected individuals is very limited, as most large BCG efficacy studies were done prior to the onset of the HIV pandemic. In HIV-infected adults, BCG vaccination has not been shown to have a statistically significant effect in preventing pulmonary or disseminated TB disease [16]. A few studies have been reported in children. A Zambian retrospective case-control study reported no protective effect of BCG in 116 TB cases and 154 controls without TB; by contrast, a 59% protective effect (OR 0.41, 95% CI 0.18–0.92) of BCG vaccination was found in HIV-uninfected children [17]. Another retrospective study in Argentina found that BCG-vaccinated HIV-infected children did not have a significantly lower incidence of TB compared to unvaccinated children during a 12-year followup period (Abstract WeOa0104, third IAS Conference on HIV Pathogenesis and Treatment, 2005, [18]). 

Such retrospective epidemiological studies are limited by potential bias due to nonstandardised criteria for the diagnosis of TB, potential selective BCG vaccination practices, and poor documentation of BCG vaccination or the use of a BCG scar to indicate BCG vaccination which has been reported to be less frequent in HIV-infected compared to HIV-uninfected children [19].

The prospect of prospective randomized trials evaluating the safety and efficacy of BCG vaccination in HIV-exposed and HIV-infected infants and children is complicated by the factors described: lack of a clinical or immune correlate of BCG-induced protection, evidence of efficacy against disseminated forms of TB in HIV-uninfected children, difficulties in diagnostic criteria for TB disease in children, and the fact that the HIV status of infants is not known at birth which is when BCG vaccine is administered in developing countries. An additional area of uncertainty is the effect of early initiation of antiretroviral therapy (ART) in infants on BCG-induced immunity, and the potential role of delayed BCG vaccination until after ART or revaccination approaches.

## 3. Safety of BCG Vaccine

The normal evolution of the local skin reaction following intradermal vaccination with BCG includes the development of an erythematous macule (3 weeks after vaccination), formation of a papule (by 6 weeks), shallow ulcer (by 10 weeks), and healing (by 14 weeks). 

Prior to the HIV pandemic, BCG had a good safety record with mostly minor reactions reported and serious adverse events generally restricted to infants with primary immunodeficiency disorders including severe combined immunodeficiency (SCID), chronic granulomatous disease (CGD), DiGeorge syndrome, IFN-gamma receptor deficiency, and IL-12 deficiency [19]. Local adverse events in infants have been estimated to occur in <0.04% of vaccine recipients and disseminated BCG disease in <0.002% of vaccine recipients [20, 21]. 

More serious BCG disease has been described in HIV-infected infants and even in an adult HIV-infected individual, 30 years after vaccination [22]. A subsequent multicentre study showed that the risk of disseminated BCG disease in immunosuppressed HIV-infected adults who received BCG vaccination during infancy was very low [23]. However, the risk of both local and disseminated BCG disease among HIV-infected infants and children has emerged as a significant public health concern. 

Two sentinel studies from South Africa, although retrospective case series, suggested a much higher prevalence of culture-confirmed distant or disseminated BCG disease among immunosuppressed HIV-infected infants not yet on ART than was previously recognized. The first study highlighted one important explanation for underrecognition of BCG disease. Laboratory culture of respiratory secretions (including gastric lavage samples) for tuberculosis identifies *M. tuberculosis* complex (which includes *M. tuberculosis* and *M. bovis* strains amongst others), and further speciation is not routinely performed. In this study, researchers performed further speciation of the *M. tuberculosis* complex in 183 cultured isolates from 49 HIV-infected children diagnosed with “tuberculosis” [24]. Danish strain *M. bovis* BCG was isolated from five infants all of whom were severely immunosuppressed. Within the group of five infants, four had axillary lymphadenitis ipsilateral to the BCG vaccination site, two had evidence of pulmonary BCG disease, and two had regional and pulmonary BCG disease. 

The second study was a hospital-based retrospective review of culture-confirmed BCG disease in children <13 yrs of age diagnosed over a three-year period [25]. Among 25 children, 88% had local disease, 32% had distant or disseminated disease, and 20% had both. Seventeen of the 25 children were HIV-infected, and two had other immunodeficiencies. All eight children with distant or disseminated disease were severely immunodeficient (six were HIV infected), and the mortality rate in this group was 75%.

Mathematical modeling approaches estimate the risk of disseminated BCG in HIV-infected infants in the South African context to be between 329 and 417 per 100 000 vaccine recipients, assuming 95% BCG coverage, HIV prevalence of 12.4–15.4% pregnant women, and a vertical HIV transmission rate of 5% [26]. This is extremely high. 

Another important observation is that BCG is a relatively common association with the immune reconstitution inflammatory syndrome (IRIS) among infants and young children initiating ART. IRIS refers to the occurrence of a paradoxical infectious or inflammatory condition in a patient recovering from severe immune deficiency [27]. The “unmasking” of TB following the initiation of ART is one of the commonest forms of IRIS described in adults whereas BCG-IRIS presenting as a “paradoxical” form of the syndrome has been reported in 3–14.8% of children starting ART in South African and Thai cohorts [28–30]. The usual clinical manifestation of BCG-IRIS is with inflammatory ipsilateral axillary or regional lymphadenitis, with or without inflammation or abscess formation at the vaccination site. Younger age and higher baseline HIV viral load have been identified as risk factors for the development of BCG-IRIS [29]. Although suspected distant or disseminated disease, particularly involving the lungs or bone, has been described in the context of BCG-IRIS, it is unclear whether dissemination may occur early after vaccination at birth but only manifest clinically following ART initiation at a few months of age. 

Should BCG vaccination at birth be delayed in HIV-exposed infants and avoided altogether in HIV-infected infants? 

As discussed above, clinical and immunological evidence for the protective efficacy of BCG vaccination in HIV-infected infants is lacking, and there are serious concerns regarding the safety of BCG in HIV-infected infants both before and after ART initiation. In 2007, the Global Advisory Committee on Vaccine Safety (GACVS) of the World Health Organization (WHO) published revised recommendations contraindicating BCG vaccination in children with HIV infection [31]. 

A more relevant consideration is whether BCG vaccine should be given to all infants including infants born to HIV-infected mothers at birth or whether selective deferral of BCG vaccination should be instituted for HIV-exposed infants until the HIV infection status is definitively established by virological testing at around 6 weeks of age. Increasing coverage of measures to reduce mother-to-child transmission of HIV (including maternal and infant preventive antiretroviral medicines or maternal antiretroviral therapy, and safe infant feeding) results in the majority of infants born to HIV-infected mothers ultimately being uninfected. These infants are expected to benefit from BCG vaccination without the risk of disseminated BCG disease reported in HIV-infected infants. Delaying BCG vaccination from birth to 10 weeks of age may result in an enhanced memory CD4 T cell response including polyfunctional T cells expressing IFN-*γ*, TNF-*α*, and IL-2 when measured at 1 year of age in HIV-uninfected infants [32]. This may also be applicable to HIV-exposed uninfected infants, but further laboratory and clinical studies are needed. 

There is a concern that selective vaccination deferral practices could threaten the benefits of high BCG coverage rates as a result of HIV-exposed but uninfected children missing followup vaccination after 6 weeks of age. The BCG Working Group of the Child Lung Health Section of the International Union Against TB and Lung Disease has published a consensus statement on relevant criteria to be considered by HIV/TB programmes considering introduction of selective BCG vaccination deferral [33]. These include high uptake of maternal HIV testing coupled with effective prevention of mother-to-child transmission (PMTCT) strategies, including maternal ART; early virological diagnosis of HIV infection in infants coupled with institution of ART; coordination of PMTCT, vaccination and TB programmes to minimise loss to followup, implement alternative TB preventive strategies, and deliver successful vaccination following selective nonvaccination at birth. Current implementation of selective vaccination strategies is not feasible in most countries that are highly endemic for HIV and TB, and universal BCG vaccination of infants is likely to continue for some time to come.

## 4. Other Ways of Protecting HIV-Infected Infants against TB

 Early initiation of ART (particularly before 12 weeks of age) in HIV-infected infants regardless of clinical or CD4 count criteria significantly reduces mortality and TB incidence [34] and may reduce the frequency and severity of BCG-IRIS, probably as a result of immune preservation (Abstract 600, fourteenth Conference on Retroviruses and Opportunistic Infections, 2008, [35]). 

Routine pre-exposure isoniazid prophylaxis for all HIV-infected infants (<12 months of age) is not currently recommended by WHO following the results of a double-blind randomized placebo-controlled trial involving 452 infants that showed no benefit (38, Abstract G2-1346a, 48th Interscience Conference on Antimicrobial Agents and Chemotherapy 2008, [36]). A previously reported study showed significant reductions in mortality and incidence of TB among HIV-infected infants and children receiving INH prophylaxis [37]. The recent WHO guidelines recommend six months of isoniazid preventive therapy (IPT) for HIV-infected children of any age with exposure to an infectious TB source case after exclusion of TB disease and six months of IPT for HIV-infected children >12 months of age who are not known to be exposed to TB [38]. 

Administration of BCG vaccine to HIV-infected infants or young children after initiation of ART and attaining some degree of immune reconstitution is another interesting approach that has not yet been studied in any detail. However, in view of the safety concerns with live vaccines in general and BCG in particular among HIV-infected or otherwise immunocompromised individuals and the revised WHO recommendation to avoid BCG in HIV-exposed infants, it seems unlikely that BCG will undergo prospective evaluation in HIV-infected infants or children. 

New BCG vaccines hold considerable promise in the medium to longer term and may ultimately address the questions surrounding efficacy and safety in a sustainable manner. Advances in genetic technology and sequencing of the *M. tuberculosis* (*M. tb*) genome during the 1990s have accelerated the development of new candidate TB vaccines, and five have progressed to the stage of Phase 1 human trials. Since HIV-infected individuals carry such a large proportion of the overall burden of TB disease, an important characteristic of the ideal TB vaccine is safety, immunogenicity, and efficacy in preventing TB in individuals with HIV infection or AIDS, including children. Live attenuated mycobacterial vaccines, such as BCG, are likely to be superseded by recombinant vectored vaccines or subunit vaccines [39]. 

New vaccines need to be tested at different phases of the natural history of TB infection and disease development. Vaccination strategies include preinfection vaccination administered at or shortly after birth (as with BCG) or as a booster following BCG at the time of birth. This approach is well suited to high burden TB environments, and the boosting approach has the advantage of potentially improving the immunogenicity and efficacy of BCG while avoiding the ethical dilemma of avoiding BCG altogether. The disadvantage of the boosting approach following BCG is that the safety concerns with BCG use particularly in HIV-infected infants remain. 

Another approach is postinfection vaccination following evidence of natural infection with *M. tb* with the aim of enhancing or boosting immunity and preventing progression to TB disease. This approach, provided the vaccine is safe, would be particularly relevant to HIV-infected individuals including young children with their high rates of TB exposure and infection, and increased risk of progression to TB disease, including severe forms of extrapulmonary disease. Two recombinant vectored vaccines (MVA85A and AERAS-402) are being investigated for use as boosting vaccines in BCG-primed individuals and for postinfection vaccination. These are the most likely candidate vaccines and vaccination strategies around which ethically acceptable clinical trials among infants and children, including those who are HIV exposed or HIV infected, could be designed [39]. 

Few, if any, of the leading candidate TB vaccines have reached clinical studies in HIV-infected children as yet, however, these two recombinant vectored vaccines have made considerable progress along the vaccine development pipeline. MVA85A consists of modified vaccinia Ankara (MVA) genetically engineered to express *M. tb* antigen 85A [40]. MVA-based vaccines have a good safety record following extensive use during the smallpox eradication era and in candidate HIV vaccine studies [41, 42]. Safety and immunogenicity data for MVA85A in HIV-infected, TB-infected, and HIV-TB coinfected adults and in healthy children and infants is awaited (Hawkridge A. in Proceedings of Tuberculosis Vaccines for the World Conference, 2006, see [43]). The AERAS-402 vaccine uses a serotype 35 adenovirus unable to replicate but modified to express a fusion protein of three *M. tb* antigens (85A, 85B, and TB10.4). It was developed to be used as a boosting vaccine in individuals primed with BCG and has entered human clinical studies [39].

A subunit vaccine, Mtb72F comprises a fusion protein of two immunogenic *M. tb* proteins combined with an adjuvant. A modification, the M72 vaccine, was shown to be safe and immunogenic in healthy adult volunteers, although more reactogenic in individuals previously exposed to *M. tb.* Clinical trials are ongoing in Europe and South Africa [39]. 

Challenges to the development of new TB vaccines include the limited understanding of protective immune responses against TB, difficulties in identifying latent infection and differentiating infection from disease, difficulty in defining clinical endpoints of trials, and the need for improved surveillance of TB-related morbidity and mortality in the context of large Phase 3 vaccine trials carried out over many years [39]. 

Another significant challenge relates to ethical issues surrounding the introduction of a new TB vaccine in the context of an existing though very imperfect vaccine (BCG) which is routinely administered to all newborn infants in TB endemic countries. It is likely that few if any countries have successfully implemented the WHO recommendation to avoid BCG vaccination in HIV-exposed newborn infants until the definitive HIV status can be established at 6 weeks of age. From an ethical perspective, vaccine trials involving infants and children that exclude BCG vaccination in their design need to consider the inclusion of alternative TB preventive measures, such as the provision of isoniazid preventive therapy and routine early initiation of ART for HIV-infected infants and children, as these are likely to become standard-of-care interventions. Since new TB vaccines directed at HIV-infected individuals are unlikely to be live attenuated vaccines, IPT is unlikely to interfere with measures of vaccine immunogenicity but may reduce the risk of TB-infected individuals developing active TB disease. Immune preservation or restoration due to ART, however, is likely to increase vaccine immunogenicity as compared to HIV-infected individuals not receiving ART. The moral imperative to incorporate such interventions into vaccine trials involving HIV-infected infants and children should override other considerations.

## 5. Conclusion

BCG vaccination of HIV-infected infants is of uncertain efficacy and is associated with significant safety concerns in untreated infants and in those on ART. The diagnosis and management of BCG disease is complex, leading to underrecognition and suboptimal care in resource-limited settings. Universal BCG immunization at birth is associated with high rates of coverage whereas selective deferral of BCG immunization until HIV infection status of infants is established risks significantly reduced BCG coverage rates amongst the large proportion of HIV-exposed but uninfected infants. Universal early initiation of ART in HIV-infected infants may currently be the most effective approach to reducing the risk of both BCG disease and TB disease in this group. Isoniazid preventive therapy for TB-exposed infants and older children is an important additional TB prevention strategy while new BCG vaccines with better safety and efficacy profiles are under investigation.
